# Bilateral Optic Neuritis Following Acute Glyphosate Inhalation: A Case Report

**DOI:** 10.3390/neurolint18020039

**Published:** 2026-02-23

**Authors:** Roberta Grasso, Elena Carapelle, Maria Eva Terracciano, Giuseppe Raunich, Antonio Turco, Luigi Longo, Ciro Mundi

**Affiliations:** Neurology Unit, Department of Neurosciences, Policlinico Riuniti di Foggia, 71100 Foggia, Italy; elena.carapelle@unifg.it (E.C.); meterracciano@ospedaliriunitifoggia.it (M.E.T.);

**Keywords:** optic neuritis, glyphosate, neurotoxicity, corticosteroids, toxic optic neuropathy

## Abstract

Background: Bilateral optic neuritis is a rare condition generally associated with inflammatory, demyelinating, or toxic causes. Its association with glyphosate exposure has rarely been documented. Case Presentation: A 66-year-old man with hypertension, hypercholesterolemia, and hypothyroidism developed rapid bilateral vision loss within hours after acute inhalational exposure to glyphosate during agricultural work. MRI showed bilateral optic nerve hyperintensity consistent with optic neuritis. Cerebrospinal fluid and serum anti-NMO antibody tests were negative, while visual evoked potentials demonstrated increased latencies. High-dose corticosteroid therapy led to progressive clinical improvement. At follow-up, MRI revealed no new lesions and the patient experienced near-complete visual recovery. Conclusion: This case suggests a possible link between acute glyphosate exposure and reversible bilateral optic neuritis. Early recognition and corticosteroid therapy may support full functional recovery even in severe presentations.

## 1. Introduction

Bilateral optic neuritis represents a rare clinical condition compared with the more common unilateral form and is often associated with a more severe course and a potentially less favorable visual prognosis. In clinical practice, simultaneous involvement of both optic nerves constitutes an important diagnostic red flag, as it significantly broadens the spectrum of possible etiologies compared with isolated unilateral optic neuritis. In particular, acute bilateral presentation requires a more thorough and timely evaluation in order to identify potentially reversible but rapidly progressive causes [1,2].

Unlike optic neuritis typically associated with multiple sclerosis, which in most cases presents unilaterally, bilateral optic neuritis is more frequently related to atypical conditions, such as systemic inflammatory diseases, infections, metabolic disorders, or toxic exposures. In this context, simultaneous bilaterality often suggests a systemic pathogenic mechanism rather than a focal demyelinating process [2]. Moreover, acute bilateral onset may be accompanied by more severe visual loss, with a greater functional impact and impairment of the patient’s quality of life.

From an epidemiological perspective, the true incidence of bilateral optic neuritis is difficult to estimate, partly due to the heterogeneity of underlying causes and the different definitions used in clinical studies. However, most available case series agree in considering it an uncommon manifestation, often reported in association with non-demyelinating conditions. For this reason, bilateral optic neuritis requires a systematic and multidisciplinary diagnostic approach, including not only neuroimaging and immunological investigations but also a careful medical history, with particular attention to environmental and occupational factors.

Early recognition of bilateral optic neuritis and its possible underlying etiology plays a crucial role, as timely treatment may significantly influence visual outcome. In particular, in forms secondary to inflammatory or toxic processes, early therapeutic intervention may promote substantial functional recovery and prevent permanent damage to the optic nerve [3,4].

Bilateral optic neuritis poses a significant diagnostic challenge, as it may represent the initial manifestation of a wide range of pathological conditions. Unlike the unilateral form, frequently associated with multiple sclerosis, simultaneous bilateral presentation requires a systematic evaluation of possible demyelinating, infectious, inflammatory, vascular, metabolic, and toxic etiologies [5].

Among demyelinating causes, neuromyelitis optica and neuromyelitis optica spectrum disorders (NMOSD), including aquaporin-4 antibody-associated disease and myelin oligodendrocyte glycoprotein (MOG) antibody-associated disease, represent conditions of primary importance in the differential diagnosis of bilateral optic neuritis. These disorders are often characterized by bilateral involvement, greater clinical severity, and a higher risk of relapse compared with multiple sclerosis-associated optic neuritis. Nevertheless, specific clinical, radiological, and laboratory features allow appropriate diagnostic orientation and differentiation among these conditions [6,7].

Infections represent another relevant group of causes of bilateral optic neuritis. Viral agents such as herpes simplex, varicella zoster, Epstein–Barr virus, HIV, and influenza viruses may involve the optic nerve either through direct invasion or through post-infectious immune-mediated mechanisms. Bacterial infections, including syphilis, tuberculosis, and brucellosis, should also be considered, particularly in the presence of systemic signs or specific risk factors [7,8,9].

Other causes include systemic inflammatory diseases and vasculitides, such as giant cell arteritis and other autoimmune vasculitic disorders, which may compromise optic nerve perfusion or cause direct inflammatory damage. In addition, paraneoplastic syndromes and hematological malignancies, such as lymphomas and leukemias, may rarely present with bilateral optic neuritis [10,11].

Toxic optic neuropathies represent a relevant chapter in the differential diagnosis, particularly in cases with acute or subacute onset. Substances such as methanol, ethylene glycol, industrial solvents, and certain drugs, including antibiotics, chloroquine, hydroxychloroquine, amiodarone, tamoxifen, and interferons, are known for their potential neurotoxic effects on the optic nerve. In this context, identification of a recent toxic exposure plays a fundamental role in guiding diagnosis and enabling timely treatment [12].

The optic nerve is particularly vulnerable to toxic and metabolic insults due to its specific anatomical and functional characteristics. It is a tissue with high energy demand, rich in mitochondria and with limited regenerative capacity, making it especially sensitive to conditions that interfere with cellular metabolism, mitochondrial function, and oxidative homeostasis. For these reasons, numerous toxic agents, both environmental and pharmacological, are known to cause optic neuropathies through direct or indirect mechanisms [13].

Toxic optic neuropathies are generally characterized by progressive visual loss, often bilateral and symmetrical, which may be accompanied by visual field defects and electrophysiological signs of axonal damage. Although onset is frequently subacute, in some cases acute exposure to neurotoxic substances may lead to rapid deterioration of visual function. Recognition of these forms is particularly important, as withdrawal of the causative agent and early initiation of appropriate treatment may significantly influence visual prognosis [14].

In recent years, increasing attention has been directed toward the potential neurological impact of environmental exposures, including pesticides and herbicides widely used in agricultural settings. Although these compounds are generally considered safe when used according to guidelines, experimental and preclinical evidence suggests that some substances may exert neurotoxic effects through multiple mechanisms, including oxidative stress, mitochondrial dysfunction, disruption of the blood–brain barrier, and activation of neuroinflammatory processes. In this context, acute inhalational exposure represents a potential route of systemic absorption, with possible effects on both the central and peripheral nervous systems [15,16].

In light of these considerations, identification of a possible environmental exposure plays a central role in the diagnostic evaluation of patients with acute or atypical bilateral optic neuritis. A thorough environmental and occupational history not only helps orient the diagnostic pathway but may also facilitate timely therapeutic intervention, potentially limiting neurological damage and improving functional outcome.

## 2. Case Presentation

This manuscript is prepared according to CARE guidelines, which were added to the Supplementary Materials.

A 66-year-old man with a history of hypertension, hypercholesterolemia, and hypothyroidism presented with progressive bilateral visual loss. He had no previous neurological or autoimmune diseases and no exposure to drugs known for their neurotoxicity.

On 21 April 2025, after several days of flu-like symptoms, the patient performed agricultural activities and was accidentally exposed by inhalation to vapors and aerosols of a glyphosate-based herbicide, without adequate respiratory protection. Within a few hours, he developed frontal headache and bilateral blurred vision. Over the following four days, visual disturbances significantly worsened.

On 25 April, he presented to the emergency department, where brain computed tomography and ophthalmological evaluation showed no relevant abnormalities. The following day, visual impairment became severe. For this reason, he returned to the emergency department and was admitted to the neurology ward.

At hospital admission on 26 April, neurological examination revealed visual acuity limited to light perception in both eyes. Pupillary reflexes were slowed, with a relative afferent pupillary defect. Ocular movements were preserved, and no other focal neurological deficits were present. During hospitalization, several instrumental examinations were performed.

**Figure 1:**
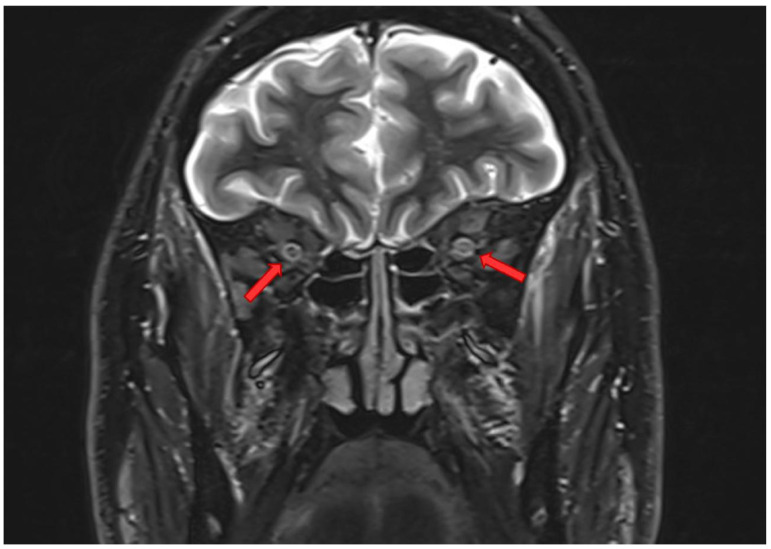
FLAIR MRI showing bilateral hyperintense signal of the optic nerves, consistent with acute bilateral optic neuritis. Arrows indicate the Hyperintense and swollen intracanicular segments of both optic nerves.

Brain magnetic resonance imaging showed bilateral T2 hyperintensities of the optic nerves, predominantly involving the intraorbital and intracanalicular segments (Figure 1). Cerebrospinal fluid examination was normal, and anti-aquaporin-4 antibodies were negative. Visual evoked potentials showed markedly prolonged latencies. Electroneurography revealed a sensory-motor polyneuropathy of the lower limbs.

**Figure 2:**
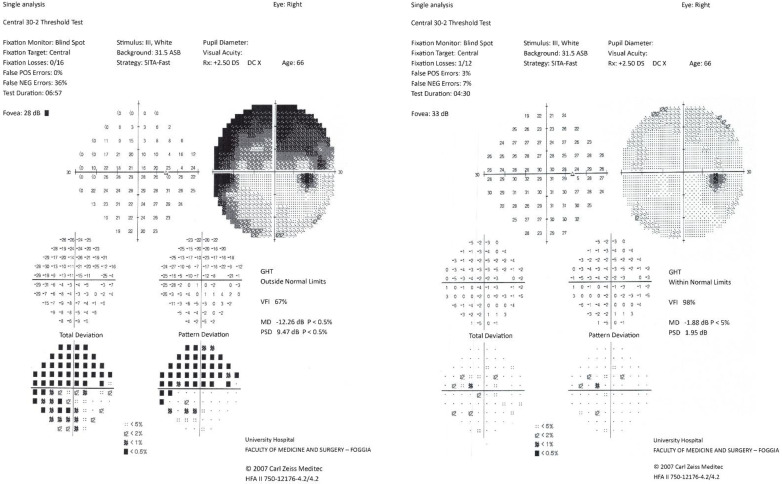
Humphrey 30-2 visual field of the right eye at presentation and follow-up. The initial test shows diffuse retinal sensitivity depression consistent with acute optic neuritis; the follow-up demonstrates significant recovery after corticosteroid therapy.

**Figure 3:**
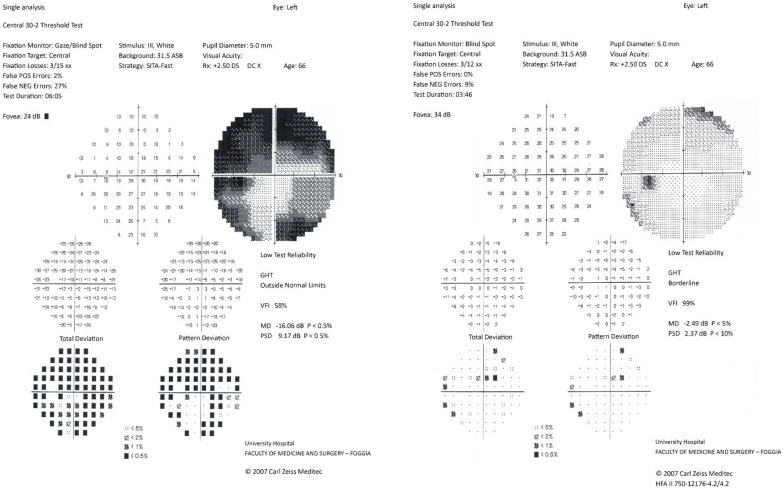
Humphrey 30-2 visual field of the left eye at presentation and follow-up. The initial test shows diffuse retinal sensitivity depression consistent with acute optic neuritis; the follow-up demonstrates marked improvement and near-complete restoration of the visual field.

The patient was treated with high-dose intravenous corticosteroids (methylprednisolone 5 g IV), with progressive visual improvement over the following days. Follow-up magnetic resonance imaging performed on 12 June 2025, showed no new lesions and no contrast enhancement of either optic nerve. Humphrey visual field testing documented marked improvement, and in the following months the patient achieved almost complete visual recovery. Visual field examinations performed during hospitalization and several weeks later showed complete recovery (Figure 2 and Figure 3).

## 3. Discussion

This case highlights a rare presentation of bilateral optic neuritis temporally associated with acute inhalation of glyphosate. The rapid onset of symptoms after exposure, the good response to corticosteroids, and the exclusion of demyelinating, autoimmune, and ischemic etiologies suggest a toxic-inflammatory mechanism, as described for other toxic agents.

Several studies suggest that glyphosate may increase the production of reactive oxygen species, reduce intracellular antioxidant systems (glutathione, superoxide dismutase, catalase), and induce neuronal oxidative damage, which could be consistent with axonal injury leading to bilateral optic neuropathy. In addition, glyphosate may be involved in disruption of the mitochondrial respiratory chain, reducing ATP production and inducing neuronal apoptosis. This mechanism may be particularly relevant for the optic nerve, which is a tissue with very high energy demand [17].

Furthermore, glyphosate may increase blood–brain barrier permeability, facilitating the passage of inflammatory mediators and promoting edema of nervous tissue. This mechanism could explain the acute onset of symptoms and the favorable response to corticosteroid therapy. Microglial activation with increased levels of tumor necrosis factor alpha, interleukin-1 beta, and interleukin-6 may also occur, leading to a central inflammatory response. Associations with peripheral sensory-motor neuropathies, electrophysiological abnormalities, and systemic nerve damage have also been reported [18].

Bilateral optic neuritis is rare and often associated with atypical causes. In our patient, the absence of previous similar episodes or other neurological symptoms, the absence of demyelinating lesions on both brain and spinal cord magnetic resonance imaging, normal cerebrospinal fluid findings, and negative anti-AQP4 antibodies excluded multiple sclerosis and other demyelinating diseases such as neuromyelitis optica spectrum disorders, including anti-MOG disease and seronegative NMOSD. Ischemic optic neuropathy was unlikely due to simultaneous bilateral onset and absence of signs of systemic vasculitis [19]. Furthermore, the negative history for use of drugs with known toxic activity or exposure to other substances, together with negative neoplastic markers, led to the most probable diagnostic hypothesis of toxic exposure to glyphosate.

Glyphosate has indeed been implicated in neurotoxic phenomena, including peripheral neuropathy. Mechanisms such as oxidative stress, mitochondrial dysfunction, excitotoxicity, and blood–brain barrier disruption may also affect the optic nerves. The presence of sensory-motor polyneuropathy strengthens the hypothesis of systemic neurological toxicity. Improvement after corticosteroid therapy suggests that inflammatory edema played a relevant role in the pathogenesis and highlights the importance of rapid diagnosis and timely treatment for possible clinical recovery.

This case underlines the importance of an accurate environmental exposure history in patients with acute bilateral visual loss and draws attention to the possible neuro-ophthalmological effects of glyphosate.

Despite the strong clinical suspicion of an association between acute glyphosate exposure and bilateral optic neuritis, this case presents some limitations that should be acknowledged. First, it represents a single clinical observation, which does not allow a definitive causal relationship between exposure and the neurological manifestation to be established. In addition, it was not possible to quantify biological levels of glyphosate or its metabolites, which could have further strengthened the etiopathogenetic hypothesis. However, the close temporal relationship between the exposure event and symptom onset, the systematic exclusion of other known causes of bilateral optic neuritis, and the favorable response to corticosteroid therapy support the plausibility of a toxic-inflammatory mechanism. In this context, the present case contributes to expanding the limited available literature on the possible neuro-ophthalmological manifestations associated with glyphosate exposure.

## 4. Conclusions

This case documents a severe bilateral optic neuritis following acute inhalation of glyphosate, with almost complete recovery after corticosteroid therapy. Clinicians should consider environmental exposures in atypical forms of optic neuritis, particularly in patients presenting with acute bilateral visual loss in the absence of clear features suggestive of demyelinating or autoimmune etiologies.

The close temporal relationship between exposure and symptom onset, the exclusion of alternative causes through extensive diagnostic evaluation, and the favorable response to high-dose corticosteroid therapy support the hypothesis of a toxic-inflammatory mechanism affecting the optic nerves. In this context, glyphosate-related neurotoxicity may involve oxidative stress, mitochondrial dysfunction, blood–brain barrier disruption, and neuroinflammatory processes, as suggested by experimental and preclinical studies.

Early recognition and timely treatment may prevent permanent visual disability and significantly improve functional outcomes. This case further highlights the importance of obtaining a detailed environmental and occupational history as an integral part of the diagnostic work-up in patients with atypical optic neuritis.

However, a definitive causal relationship between glyphosate exposure and optic neuritis cannot be established on the basis of a single observation, so further clinical and experimental studies are needed to clarify glyphosate neurotoxicity and to better define its possible role in optic nerve involvement.

## Data Availability

Data supporting the findings of this report are available from the corresponding author upon request.

## Supplementary Materials

The following supporting information can be downloaded at: https://www.mdpi.com/article/10.3390/neurolint18020039/s1, Table S1: CARE Checklist.
