# Supplementary material for: Bilateral Optic Neuritis Following Acute Glyphosate Inhalation: A Case Report
Source: Neurol Int. 2026 Feb 23;18(2):39. doi: 10.3390/neurolint18020039 (PMC12942672; doi:10.3390/neurolint18020039)
Supplement: Supplementary file 1 [file neurolint-18-00039-s001.zip › neurolint-4070760-supplementary.pdf]

CARE case report guidelines

CAREChecklistofinformationtoincludewhenwritingacasereport

(cc) BY-NC-ND

Topic	Item	Checklistitemdescription	ReportedonLine
**Title**	**1**	Thediagnosisorinterventionofprimaryfocusfollowedbythewords“casereport”..	Title________________
**KeyWords**	**2**	2to5keywordsthatidentifydiagnosesorinterventionsinthiscasereport,including"casereport"	Keyword______________
**Abstract (noreferences)**	**3a**	Introduction:Whatisuniqueaboutthiscaseandwhatdoesitaddtothescientificliterature?...	Abstract______________
	**3b**	Mainsymptomsand/orimportantclinicalfindings..	Abstract______________
	**3c**	Themaindiagnoses,therapeuticinterventions,andoutcomes	Abstract______________
	**3d**	Conclusion—Whatisthemain“take-away”lesson(s)fromthiscase?	Introduction___________
**Introduction**	**4**	Oneortwoparagraphssummarizingwhythiscaseisunique(**mayinclude**references)	____________________
**PatientInformation**	**5a**	De-identifiedpatientspecificinformation	Case presentation_____
	**5b**	Primaryconcernsandsymptomsofthepatient..	Case presentation_______
	**5c**	Medical,family,andpsycho-socialhistoryincludingrelevantgeneticinformation	Case presentation
	**5d**	Relevantpastinterventionswithoutcomes..	Case presentation
Case presentation			
**Clinical Findings**	**6**	Describesignificantphysicalexamination(PE)andimportantclinicalfindings..	Case presentation
**Timeline**	**7**	Historicalandcurrentinformationfromthisepisodeofcareorganizedasatimeline...	Case presentation
**Diagnostic Assessment**	**8a**	Diagnostictesting(suchasPE,laboratorytesting,imaging,surveys)...	Case presenation
	**8b**	Diagnosticchallenges(suchasaccess to testing,financial,or cultural)...	case presenattion_____
	**8c**	Diagnosis (including other diagnoses considered)...	Case presentation_______
	**8d**	Prognosis(suchasstaginginoncology)whereapplicable	Case presentation
**Therapeutic Intervention**	**9a**	Typesoftherapeuticintervention(suchaspharmacologic,surgical,preventive,self-care)..	Case presentation_____
	**9b**	Administrationoftherapeuticintervention(suchasdosage,strength,duration)	Case presentation_____
	**9c**	Changesintherapeuticintervention(withrationale)..	NA________________
**Follow-upand Outcomes**	**10a**	Clinicianandpatient-assessedoutcomes(ifavailable)..	Case presentation_____
	**10b**	Importantfollow-updiagnosticandothertestresults...	Follow up___________
	**10c**	Interventionadherenceandtolerability(Howwasthisassessed?)	Follow up___________
	**10d**	Adverseandunanticipatedevents	Follow up___________
**Discussion**	**11a**	AscientificdiscussionofthestrengthsANDlimitationsassociated with thiscasereport	Discussion___________
	**11b**	Discussionofthe relevantmedicalliterature**withreferences**..	Discussion___________
	**11c**	Thescientificrationaleforanyconclusions(includingassessmentofpossiblecauses)..	Discussion___________
	**11d**	Theprimary“take-away”lessonsofthiscasereport(withoutreferences)inaoneparagraphconclusion	Discussion___________
**PatientPerspective**	**12**	Thepatientshouldsharetheirperspectiveinonetotwoparagraphsonthetreatment(s)theyreceived.............................	NA________________

**InformedConsent**	**13**	Didthepatientgiveinformedconsent?Pleaseprovideifrequested.....................................	**Yes** ☒ **No** ☐
